# Immunization continuity failures and measles vaccine dropout in a fragile urban setting: Evidence from Mogadishu, Somalia

**DOI:** 10.1016/j.puhip.2026.100819

**Published:** 2026-06-03

**Authors:** Ahmed Saed Hussein, Ahmed Nor Osman, Gallad Dahir Ahmed, Leila Abdi Ibrahim, Sadia Hassan Mohamed, Sadik Jama Mohamed, Anisa Ali Abdullahi

**Affiliations:** Jamhuriya University of Science and Technology, Faculty of Medicine and Health Sciences, Mogadishu, Somalia

**Keywords:** Immunization completion, Measles vaccination, Vaccine dropout, Routine immunization, Urban health, Somalia

## Abstract

**Objectives:**

Persistent measles transmission in fragile and conflict-affected settings is often linked to failure to complete multi-dose childhood immunization schedules. This study aimed to examine immunization completion and measles vaccine dropout to assess continuity of routine immunization services among children aged 12–23 months in Hodan District, Mogadishu, Somalia.

**Study design:**

Community-based cross-sectional study.

**Methods:**

A community-based cross-sectional study was conducted between May and July 2025 among 270 caregivers of children aged 12–23 months selected using multistage cluster sampling. Vaccination status was verified using immunization cards and caregiver recall. Vaccine coverage, full immunization status, and dropout rates between successive doses were calculated. Bivariate and multivariable logistic regression analyses were performed to explore factors associated with incomplete vaccination (p ≤ 0.05).

**Results:**

Full immunization coverage was 45.2% (95% CI: 39.3–51.2), with 54.8% of children incompletely vaccinated. Coverage declined across successive vaccine doses, from 75.9% for BCG to 55.9% for Penta3 and OPV3. Measles first-dose coverage was 46.3%, decreasing to 27.0% for the second dose, resulting in a high dropout rate of 41.6%. Maternal education, antenatal care attendance, immunization advice, and caregiver knowledge were associated with incomplete vaccination in bivariate analysis; however, no independent predictors remained significant after multivariable adjustment.

**Conclusions:**

Substantial immunization dropout following vaccine initiation indicates important continuity gaps within routine immunization services in this urban fragile setting. Strengthening follow-up mechanisms, caregiver communication, and defaulter-tracking systems may improve vaccine completion and reduce measles outbreak risk.

## 1 Introduction

Immunization is one of the most effective and cost-efficient public health interventions for preventing childhood morbidity and mortality caused by vaccine-preventable diseases worldwide. Over the past decades, vaccination programs have prevented millions of deaths annually and remain central to global child survival [1,2]. Global monitoring of vaccination performance relies on the WHO and United Nations Children's Fund (UNICEF) estimates of national immunization coverage, which synthesize administrative and survey data to assess progress toward universal immunization goals [3]. Despite these achievements in expanding global immunization coverage, substantial inequalities persist, particularly in low-income and fragile settings where health systems face operational and structural challenges (see Table 1, Table 2, Table 3).

**Table 1:** Sociodemographic, maternal, and child characteristics (N = 270).

Variable	Category	Frequency (%)
**Mother age group (years)**		
	17–25	88 (32.6)
	26–35	132 (48.9)
	36–47	50 (18.5)
**Marital status**		
	Single	12 (4.4)
	Married	235 (87.0)
	Divorced	23 (8.5)
**Mother education**		
	No formal education	156 (57.8)
	Primary school	57 (21.1)
	Secondary school	41 (15.2)
	University and above	16 (5.9)
**Mother occupation**		
	Unemployed	209 (77.4)
	Self-employed	49 (18.1)
	Salaried employment	12 (4.4)
**Monthly household income (USD)**		
	<100	17 (6.3)
	100–200	57 (21.1)
	201–300	109 (40.4)
	≥301	87 (32.2)
**Child sex**		
	Male	140 (51.9)
	Female	130 (48.1)
**Total number of children**		
	1–4	151 (55.9)
	5–7	76 (28.1)
	8–10	43 (15.9)
**Birth weight**		
	<2.5 kg	63 (23.3)
	≥2.5 kg	180 (66.7)
	Don't know	27 (10.0)
**ANC attendance (visits)**		
	No ANC	56 (20.7)
	ANC 1–3 visits	94 (34.8)
	ANC ≥4 visits	120 (44.4)
**Place of delivery**		
	Health facility	209 (77.4)
	Home	61 (22.6)
**Immunization advice by health worker**		
	Yes	166 (61.5)
	No	104 (38.5)
**Knows age for completion of immunization**		
	Yes	187 (69.3)
	No	83 (30.7)
**Type of facility used**		
	Public health center	175 (64.8)
	Private clinic/hospital	95 (35.2)
**Immunization status (basic schedule)**		
	Incomplete vaccination	148 (54.8)
	Fully vaccinated	122 (45.2)

**Table 2:** Immunization coverage and dose-specific dropout rates (N = 270).

Indicator	n/N (%)	95% CI
BCG	205/270 (75.9)	70.4–80.7
OPV0	189/270 (70.0)	64.2–75.2
Penta1	180/270 (66.7)	60.8–72.1
Penta3	151/270 (55.9)	49.9–61.8
OPV3	151/270 (55.9)	49.9–61.8
Measles1	125/270 (46.3)	40.4–52.3
Measles2	73/270 (27.0)	22.0–32.7
Fully vaccinated (basic schedule)	122/270 (45.2)	39.3–51.2
Penta1–Penta3 dropout		16.1%
Measles1–Measles2 dropout		41.6%

Dropout rate = [(coverage of previous dose − coverage of subsequent dose)/coverage of previous dose] × 100.

**Table 3:** Factors associated with incomplete vaccination among children aged 12–23 months.

Variable	Category	COR (95% CI)	p-value	AOR (95% CI)	p-value
**Mother education**					
	No formal education			(Ref)	
	Primary school	1.00 (0.54–1.86)	0.996	1.13 (0.60–2.14)	0.703
	Secondary school	0.48 (0.24–0.97)	0.039	0.57 (0.28–1.18)	0.130
	University and above	0.23 (0.07–0.73)	0.013	0.37 (0.11–1.28)	0.118
**ANC attendance**					
	No ANC	(Ref)			
	ANC 1–3 visits	0.67 (0.33–1.34)	0.255	0.96 (0.44–2.08)	0.920
	ANC ≥4 visits	0.40 (0.21–0.78)	0.007	0.65 (0.31–1.38)	0.263
**Immunization advice**					
	Yes	(Ref)			
	No	2.03 (1.22–3.37)	0.006	1.70 (0.98–2.94)	0.059
**Knowledge of completion age**					
	Yes	(Ref)			
	No	1.84 (1.08–3.14)	0.025	1.41 (0.78–2.53)	0.255

Globally, failure to complete multi-dose vaccination schedules remains a major concern, contributing to the growing number of under-immunized children. The Immunization Agenda 2030 emphasizes reducing zero-dose and partially vaccinated children by strengthening equitable access, continuity of care, and follow-up mechanisms within routine immunization systems [1,4]. Measles vaccination coverage is especially sensitive to program performance because achieving herd immunity requires high coverage with two doses of measles-containing vaccine [5,6]. High dropout between the first and second measles doses has been reported globally and is considered an indicator of weaknesses in service continuity and caregiver engagement.

In the WHO African Region and broader sub-Saharan Africa, immunization completion remains uneven despite expanded vaccination programs. Systematic reviews have shown persistent vaccination dropout associated with socioeconomic disparities, maternal education, health service accessibility, and missed opportunities for vaccination [7,8]. Previous studies across low- and middle-income countries have consistently identified maternal education and awareness of vaccination schedules as important determinants of immunization completion [9]. Studies conducted in Ethiopia and other African countries demonstrate that measles second-dose dropout continues to pose a significant public health challenge, particularly in rural and underserved communities [10,11]. Missed opportunities for vaccination within health facilities further contribute to incomplete immunization even when children interact with healthcare services [[12], [13], [14]].

Fragile and conflict-affected countries experience additional barriers that disrupt routine immunization delivery. Population displacement, insecurity, limited infrastructure, and health workforce shortages reduce consistent access to vaccination services [15]. Somalia represents one of the most complex fragile health contexts globally, characterized by prolonged conflict, humanitarian crises, and large internally displaced populations [16]. Although recent reports indicate gradual progress in childhood immunization coverage, substantial gaps remain in reaching and retaining children within the vaccination schedule. Recent WHO–UNICEF estimates indicate gradual improvements in national immunization coverage in Somalia, although substantial gaps remain in ensuring full immunization and retention within the vaccination schedule [17,18].

National evidence from Somalia indicates persistent challenges related to incomplete vaccination and the presence of zero-dose and under-immunized children, particularly in inaccessible and conflict-affected areas [19,20]. Recurrent outbreaks of vaccine-preventable diseases such as measles and diphtheria further highlight weaknesses in routine immunization performance and follow-up [19,21]. While national surveys provide overall coverage estimates [22], these data often mask important subnational disparities that influence program effectiveness at district level.

Evidence specific to Mogadishu has begun to emerge. Facility-based studies have reported suboptimal immunization coverage and identified maternal and service-related factors influencing vaccination uptake among children aged 12–23 months [23,24]. Additionally, assessments of missed opportunities for vaccination in Mogadishu demonstrated that many eligible children visiting health facilities fail to receive recommended vaccines, suggesting systemic inefficiencies within service delivery [25]. However, most existing studies rely on facility-based samples and provide limited information on community-level immunization completion and dose-specific dropout patterns, particularly for measles vaccination.

Understanding vaccine dropout at community level is critical because children who initiate vaccination but fail to complete the schedule remain vulnerable to infection and contribute to outbreak transmission. WHO guidance emphasizes monitoring dropout rates as an essential indicator for strengthening routine immunization programs and improving defaulter-tracking strategies [6,26]. Despite the recognized importance of monitoring vaccination dropout, limited evidence exists explaining whether incomplete immunization in fragile urban settings reflects caregiver-related barriers or weaknesses in continuity of routine immunization services. In Somalia, available evidence largely relies on national estimates or facility-based studies, which provide limited insight into how children progress through successive vaccine doses within rapidly growing urban populations such as Mogadishu. As a result, program planners lack community-level evidence needed to understand where breakdowns occur along the immunization pathway after children initiate vaccination. This study therefore examined immunization completion and dose-specific dropout, particularly between Penta1–Penta3 and Measles1–Measles2, to assess continuity of routine immunization and identify whether gaps are primarily associated with individual or system-level factors among children aged 12–23 months in Hodan District, Mogadishu, Somalia.

## 2 Methods

A community-based cross-sectional study was conducted between May and July 2025 in Hodan District, Mogadishu, Somalia, an urban setting characterized by rapid population movement and ongoing health system constraints. The study was designed to generate programmatically relevant evidence to inform strengthening of routine immunization follow-up and service continuity at district level. Caregivers of children aged 12–23 months who had resided in the district for at least six months prior to the survey were included. This age group was selected because children are expected to have completed the basic childhood immunization schedule according to national Expanded Programme on Immunization guidelines. Caregivers who were unable to provide vaccination information or consent were excluded.

The sample size was calculated using a single population proportion formula based on a previous Somali study reporting 20% full immunization coverage among children aged 11–24 months 27, assuming a 95% confidence level and 5% margin of error. The minimum sample size was 246; after adding a 10% allowance for non-response, 270 caregiver–child pairs were included. A multistage cluster sampling approach was employed. Clusters (neighbourhoods) were selected using probability proportional to size based on district population data, followed by systematic sampling of households within each cluster. A total of 9 clusters were selected, with 30 households sampled per cluster. Data were collected through face-to-face interviews using a structured questionnaire adapted from World Health Organization immunization coverage survey tools. Information on sociodemographic characteristics, maternal health service utilization, caregiver knowledge of immunization, and child vaccination history was obtained. Vaccination status was verified using immunization cards whenever available; caregiver recall was used when cards were unavailable.

The primary outcome variable was immunization status, categorized as fully vaccinated or incompletely vaccinated according to the national basic immunization schedule. In Somalia, the first dose of measles vaccine is administered at 9 months and the second dose at 15 months according to the national immunization schedule. A child was considered fully vaccinated if they received all recommended vaccines according to the national immunization schedule by 12 months of age. Children who missed one or more doses were classified as incompletely vaccinated. Children who had not received any vaccine were categorized as zero-dose children, and coverage for the second dose of measles vaccine was calculated only among those who were age-eligible according to the national immunization schedule, with those not yet eligible excluded from the denominator. Independent variables included maternal sociodemographic characteristics, antenatal care attendance, place of delivery, receipt of immunization advice, caregiver knowledge of immunization completion age, and selected health service utilization factors.

Data were entered, cleaned, and analyzed using Stata version 14.0 (StataCorp LLC, College Station, TX, USA). Descriptive statistics summarized participant characteristics and vaccination coverage. Vaccine-specific coverage proportions with 95% confidence intervals were calculated, and dropout rates were estimated between Penta1–Penta3 and Measles1–Measles2 doses. Bivariate and multivariable logistic regression analyses were conducted to explore factors associated with incomplete vaccination, with adjusted odds ratios and 95% confidence intervals reported. Variables with p < 0.25 in bivariate analysis were included in the multivariable logistic regression model. Multicollinearity was assessed using variance inflation factors (VIF), and no significant multicollinearity was observed. Model fit was assessed using the Hosmer–Lemeshow goodness-of-fit test (p = 0.72), indicating good model fit. Missing data were minimal, with fewer than 5% of observations missing for any variable, and were handled using complete case analysis.

Ethical approval was obtained from the Institutional Review Board of Jamhuriya University of Science and Technology, Mogadishu, Somalia. Written informed consent was obtained from all participants prior to data collection, and confidentiality of participant information was maintained throughout the study.

## 3 Results

### 3.1 Sociodemographic and maternal characteristics

A total of 270 caregiver–child pairs were included in the analysis. Nearly half of the mothers were aged 26–35 years (48.9%), followed by 17–25 years (32.6%) and 36–47 years (18.5%). The majority of respondents were married (87.0%). More than half of mothers (57.8%) had no formal education, while 5.9% had university-level education. Most mothers were unemployed (77.4%).

Regarding household income, 40.4% reported earning USD 201–300 per month, and 32.2% earned ≥ USD 301. Slightly more than half of the children were male (51.9%). Over half of mothers (55.9%) had between one and four children.

Most children were born in health facilities (77.4%). Although 79.3% of mothers attended at least one antenatal care (ANC) visit, only 44.4% completed four or more visits. Immunization advice from a health worker was reported by 61.5% of mothers. Approximately 69.3% of respondents knew the recommended age for completion of childhood immunization. Public health centers were the primary source of vaccination services (64.8%).

### 3.2 Immunization coverage and dropout

Full immunization coverage according to the basic schedule was 45.2% (95% CI: 39.3–51.2), while 54.8% of children were incompletely vaccinated.

Coverage declined progressively across vaccine doses. BCG coverage was 75.9% (95% CI: 70.4–80.7), and Penta1 coverage was 66.7% (95% CI: 60.8–72.1). However, only 55.9% (95% CI: 49.9–61.8) completed the third dose of pentavalent vaccine and OPV3. Measles first-dose coverage was 46.3% (95% CI: 40.4–52.3), decreasing substantially to 27.0% (95% CI: 22.0–32.7) for the second dose.

The dropout rate between Penta1 and Penta3 was 16.1%, while dropout between Measles1 and Measles2 was markedly higher at 41.6%, indicating substantial attrition at later stages of the immunization schedule.

### 3.3 Factors associated with incomplete vaccination

In bivariate logistic regression analysis, maternal education, ANC attendance, receipt of immunization advice, and knowledge of the recommended age for immunization completion were significantly associated with incomplete vaccination.

Compared with mothers who had no formal education, those with secondary education (COR 0.48; 95% CI: 0.24–0.97) and university-level education (COR 0.23; 95% CI: 0.07–0.73) had lower odds of incomplete vaccination. Completing four or more ANC visits was also protective (COR 0.40; 95% CI: 0.21–0.78). Mothers who did not receive immunization advice had higher odds of incomplete vaccination (COR 2.03; 95% CI: 1.22–3.37), as did those who did not know the recommended age for immunization completion (COR 1.84; 95% CI: 1.08–3.14).

In multivariable logistic regression analysis adjusting for education, ANC attendance, immunization advice, and maternal knowledge, no independent predictor remained statistically significant at the 0.05 level. However, lack of immunization advice remained suggestive of an association but not statistically significant (AOR 1.70; 95% CI: 0.98–2.94; p = 0.059). Higher maternal education retained a protective direction, although the associations were attenuated after adjustment.

## 4 Discussion

This study provides community-based evidence that immunization dropout in an urban fragile setting is primarily characterized by loss of continuity after vaccination initiation rather than lack of initial access to vaccination services. Although a substantial proportion of children received early vaccines, completion declined progressively across successive doses, resulting in low full immunization coverage (45.2%) and a particularly high dropout between the first and second doses of measles vaccine (41.6%). These findings highlight persistent challenges in maintaining continuity of routine immunization services in rapidly growing urban fragile contexts [27,28]. Studies conducted in informal urban settlements in Kenya and other African contexts similarly demonstrate that access alone does not guarantee immunization completion [29]. The corresponding measles second-dose coverage of 27.0% is far below the level required to achieve herd immunity, indicating a considerable accumulation of susceptible children within the community. In densely populated urban settings such as Mogadishu, such immunity gaps increase the risk of sustained measles transmission and recurrent outbreaks despite ongoing vaccination efforts. These findings further underscore that while many children initiate vaccination, maintaining continuity through completion of the schedule remains a major challenge in this setting.

The level of full immunization observed in this study is comparable to findings reported in several low- and middle-income countries but remains below the levels required to achieve population immunity against vaccine-preventable diseases. Systematic reviews conducted across sub-Saharan Africa have similarly documented persistent gaps in immunization completion despite improvements in initial vaccine uptake [7,8]. Studies conducted in Ethiopia and other African contexts have also reported considerable measles vaccination dropout, highlighting ongoing challenges in sustaining caregiver engagement throughout multi-dose vaccination schedules [10,11]. The particularly high measles dropout observed in this study suggests that challenges occur primarily in retaining children within the immunization schedule rather than initiating vaccination. Comparable declines in coverage at later vaccine doses have been documented across sub-Saharan Africa and are commonly linked to missed opportunities for vaccination and weak defaulter-tracking systems [7,30].

In fragile and conflict-affected settings such as Somalia, immunization performance is strongly influenced by health system constraints and population mobility. Although recent national reports indicate gradual improvement in childhood immunization coverage, substantial numbers of children remain partially vaccinated or under-immunized [17,18]. Urban districts like Mogadishu experience rapid population movement, informal settlements, and service delivery interruptions that may hinder consistent follow-up for scheduled vaccinations. Previous assessments in Mogadishu have identified missed opportunities for vaccination within health facilities, suggesting that system-level factors—including inadequate counseling, limited reminder systems, and weak defaulter tracking—contribute to incomplete immunization [25].

Bivariate analysis identified associations between incomplete vaccination and maternal education, antenatal care attendance, receipt of immunization advice, and caregiver knowledge of the recommended age for immunization completion. However, these associations were not sustained after multivariable adjustment. This may partly reflect limited statistical power to detect modest effects, in addition to potential system-level influences. The findings suggest that incomplete immunization in this setting is more strongly driven by broader service delivery and programmatic challenges than by individual-level socioeconomic factors. Similar patterns have been reported in fragile health systems, where structural barriers often outweigh household-level determinants 19,20. Overall, these results highlight the need to strengthen routine immunization systems rather than relying solely on behavior-focused interventions [19,20].

High dropout between vaccine doses represents a critical program performance indicator emphasized by the World Health Organization's Essential Programme on Immunization. Monitoring and reducing missed opportunities for vaccination have been identified as effective strategies for improving coverage without requiring major expansion of services [6]. Evidence from multiple low-income settings demonstrates that strengthening follow-up mechanisms, improving caregiver communication, and integrating vaccination checks into all child health contacts can substantially reduce dropout rates [13,14].

### 4.1 Strengths and limitations

This study has several strengths. First, it employed a community-based design, allowing inclusion of children who may not regularly access health facilities and therefore providing more representative district-level estimates. Second, the analysis examined dose-specific dropout across the immunization schedule, offering programmatically relevant insights beyond overall coverage estimates.

However, some limitations should be considered. The cross-sectional design limits causal interpretation of associations. Vaccination status partly relied on caregiver recall when immunization cards were unavailable, which may introduce recall bias. Additionally, findings from a single urban district may not be generalizable to rural or nomadic populations in Somalia. The possibility of limited statistical power to detect small associations should also be considered, which may partly explain the lack of statistically significant predictors in the multivariable analysis.

### 4.2 Public health implications

The findings underscore that improving immunization outcomes in urban Somalia requires greater emphasis on vaccine completion rather than initial access alone. Strengthening reminder systems, caregiver counseling, defaulter tracing, and continuity of routine immunization services may be essential to reduce measles dropout and improve overall coverage. These actions align with Immunization Agenda 2030 priorities aimed at reducing under-immunized children and strengthening resilient immunization systems in fragile settings.

### 4.3 Conclusions

Immunization completion among children aged 12–23 months in Hodan District, Mogadishu remains suboptimal, with fewer than half of children fully vaccinated according to the basic schedule. The study identified substantial vaccine dropout, particularly between the first and second doses of measles vaccine, indicating important gaps in continuity of routine immunization services. Although several caregiver and service-related factors were associated with incomplete vaccination at the bivariate level, no independent predictors remained significant after adjustment, suggesting that broader health system and programmatic challenges may play a dominant role. Strengthening routine immunization follow-up mechanisms, improving caregiver counseling, and reducing missed opportunities for vaccination are essential to improve vaccine completion. Targeted district-level interventions focusing on retention within the immunization schedule may contribute to achieving national immunization goals and advancing Immunization Agenda 2030 priorities in fragile urban settings. Addressing vaccine dropout and improving completion of measles vaccination schedules will be critical to reducing the accumulation of susceptible children and preventing future measles outbreaks in fragile urban settings such as Mogadishu.

## Ethical statement

Ethical approval was obtained from the Jamhuriya University Research Ethics Committee (JUREC), Mogadishu, Somalia (Ref: JUREC0140/CGS354/052025; approved on 5 March 2025). Written informed consent was obtained from all participants prior to data collection, and confidentiality was maintained throughout the study.

## Author contributions

Ahmed Saed Hussein: Conceptualization, study design, data analysis, interpretation of results, and manuscript drafting.

Ahmed Nor Osman: Methodology development, supervision, and critical revision of the manuscript.

Gallad Dahir Ahmed: Data collection coordination and data management.

Leila Abdi Ibrahim: Field supervision and data acquisition.

Sadia Hassan Mohamed: Data collection and validation.

Sadik Jama Mohamed: Statistical support and results interpretation.

Anisa Ali Abdullahi: Literature review and manuscript editing.

All authors reviewed and approved the final version of the manuscript.

## Funding

This research received no specific grant from any funding agency in the public, commercial, or not-for-profit sectors.

## Declaration of competing interest

The authors declare that they have no competing interests.

## Data Availability

The datasets generated and analyzed during the current study are available from the corresponding author upon reasonable request. Data are not publicly available due to confidentiality considerations involving study participants.
